# Editorial: Advancing Treg cell therapy: a comprehensive review series

**DOI:** 10.3389/fimmu.2026.1923288

**Published:** 2026-07-27

**Authors:** Fabien Depis, Devan Moodley, Seng-Lai Tan

**Affiliations:** 1 TregShield Bio, Cambridge, MA, United States; 2 Thinking Tiger Group, Boston, MA, United States; 3 Romulus Therapeutics Pte. Ltd., Singapore, Singapore

**Keywords:** autoimmune disease (AD), cell therapy (CT), immune tolerance, inflammatory disorders, transplantation, Treg - regulatory T cell

Regulatory T cell (Treg) therapy has reached an inflection point. What began as a biological concept for restoring immune tolerance is rapidly evolving into a therapeutic platform with the potential to transform autoimmune diseases, transplantation, and inflammatory disorders. As the field advances toward clinical and commercial implementation, challenges now extend well beyond Treg biology itself. Cell sourcing, engineering, manufacturing, quality control, regulatory development, and clinical deployment have become equally important determinants of success. Yet overcoming these hurdles brings us closer to a true immunological reset. By bypassing the need to identify disease-specific antigens through localized bystander suppression while combining immune silencing with active tissue repair, Treg therapy represents a potential paradigm shift toward curative medicine.

**Figure 1:**
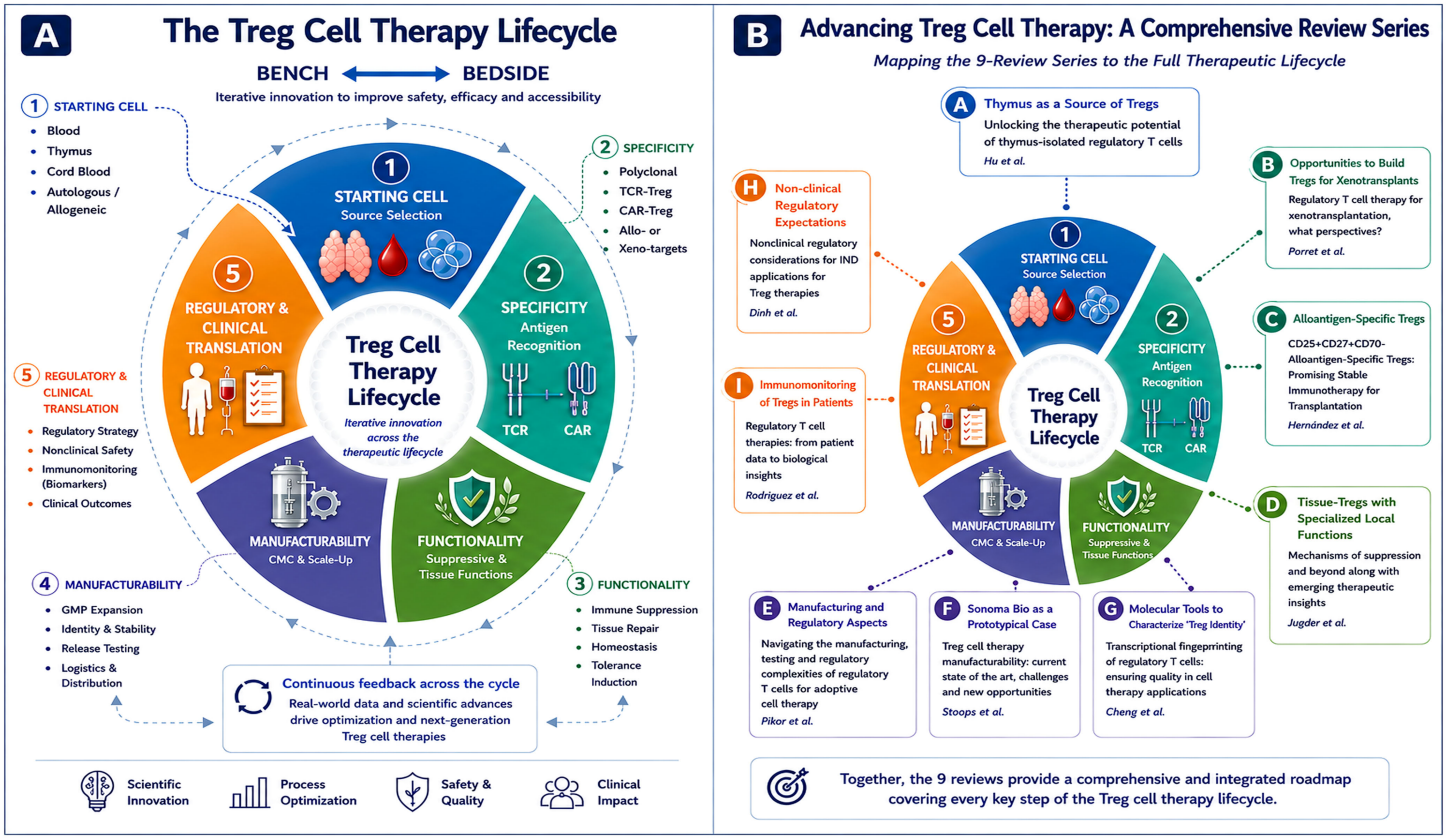
Introduces the Treg cell therapy lifecycle framework, organizing the field into five interconnected stages encompassing source selection, antigen specificity, functionality, manufacturability, and regulatory/clinical translation. **(A)** illustrates the lifecycle framework, while **(B)** maps the nine accompanying reviews across these major stages of therapeutic development.

This Research Topic is, to our knowledge, the first review collection designed to comprehensively capture the entire Treg Cell Therapy Lifecycle, spanning starting cell selection, antigen specificity, functionality, manufacturability, clinical translation, and regulatory development (**Figure 1**). Rather than focusing on a single aspect of Treg biology or therapeutic application, the Research Topic provides an integrated roadmap for translating Treg therapies from laboratory concepts into scalable and clinically impactful medicines.

The series was intentionally developed as a reference resource, bringing together complementary perspectives within a single collection for a broad audience. The nine articles collectively address the scientific, translational, manufacturing, and regulatory foundations required to advance Treg cell therapies. Reflecting the multidisciplinary nature of the field, contributors include leaders from academia, biotechnology, the pharmaceutical industry, and regulatory science. Several authors were also invited speakers at the annual Treg Summit in Boston, chaired by Dr. Fabien Depis, helping integrate current scientific, clinical, translational, and industrial perspectives into a cohesive review series.

The timing of this Research Topic is particularly appropriate. The field’s momentum is further catalyzed by the 2025 Nobel Prize in Physiology or Medicine for the pioneering discoveries of peripheral immune tolerance. By cementing Treg biology as a cornerstone of modern immunology, this global recognition highlights the urgent need to transition these foundational insights into scalable, clinical realities. At the same time, the remarkable success of chimeric antigen receptor (CAR)-T cell therapies in oncology, together with emerging reports of durable remissions in severe autoimmune diseases, has renewed enthusiasm for therapeutic strategies aimed at restoring immune tolerance rather than relying solely on chronic immunosuppression.

Adoptive Treg therapy has similarly progressed from experimental observation to clinical reality. Early studies in graft-versus-host disease (GvHD), type 1 diabetes, and transplantation established the feasibility and safety of adoptive Treg therapy. Current efforts are increasingly focused on improving functional specialization—including tissue repair and homeostatic functions in addition to immune regulation—while enhancing antigen specificity, stability, persistence, scalability, and the manufacturing and regulatory frameworks necessary for widespread clinical adoption.

A major theme emerging from this Research Topic is the development of increasingly specialized Treg products. While early clinical studies largely employed polyclonal Tregs, there is growing recognition that antigen-specific approaches may provide greater potency and precision. Cortés-Hernández et al. describe highly purified CD25^+^CD27^+^CD70^-^ alloantigen-specific Tregs that maintain lineage stability and suppressive function under inflammatory conditions. Extending this concept, Porret et al. discuss opportunities and challenges for engineered Tregs in xenotransplantation, potentially reducing dependence on conventional immunosuppression. Hu et al. review thymus-derived Tregs as an emerging cellular source with favorable biological properties and growing clinical interest. Together, these articles highlight the field’s progression toward increasingly tailored Treg products.

Another important theme is the expanding understanding of Treg biology. Once viewed primarily as suppressive cells, Tregs are now recognized as highly specialized regulators that adapt to distinct tissue environments. Jugder et al. review tissue-specific Treg functions across multiple organs, emphasizing roles in tissue repair and maintenance of local homeostasis. Complementing these biological insights, Rodrigues et al. examine how clinical data and advanced immunomonitoring technologies can better define Treg persistence, function, and therapeutic activity in patients. Together, these reviews strengthen the connection between Treg biology and clinical outcomes.

The Research Topic also illustrates the growing importance of industrializing Treg cell therapy. As Treg products move toward routine clinical application, manufacturing, quality control, and regulatory science have become central to successful translation. Unlike conventional biologics, cellular therapies require highly complex production and testing processes. Pikor et al. provide a comprehensive overview of manufacturing, quality, and regulatory challenges, while Stoops et al. draw on Sonoma Bio’s industrial experience to discuss opportunities for improving manufacturability and scalability. Complementing these perspectives, Cheng et al. present a transcriptional fingerprinting framework for molecular assessment of Treg identity, stability, and expansion status.

The Research Topic further highlights the increasingly important role of regulatory science in accelerating clinical translation. Dinh and Mihalcik review nonclinical considerations for investigational new drug applications, including pharmacology, biodistribution, safety assessment, and regulatory interactions. Their article provides practical guidance for developers navigating a rapidly evolving regulatory landscape.

The key questions facing Treg therapy are no longer whether these cells can suppress immune responses, but how best to engineer, manufacture, characterize, and deploy them to achieve durable clinical benefit. While early clinical studies established safety and feasibility, future success will depend on improving antigen specificity, tissue targeting, persistence, lineage stability, and scalable manufacturing.

Collectively, the articles in this Research Topic provide a comprehensive roadmap for advancing Treg therapies from biological concept to clinically scalable medicines. Continued innovation across each stage of the therapeutic lifecycle will be essential to realizing the full potential of immune tolerance-restoring therapies.

The Topic Editors sincerely thank all contributing authors, reviewers, and the Frontiers editorial staff for their invaluable contributions to this Research Topic.

